# In situ impedance spectroscopy of filament formation by resistive switches in polymer based structures

**DOI:** 10.1038/s41598-018-27332-1

**Published:** 2018-06-13

**Authors:** M. S. Kotova, K. A. Drozdov, T. V. Dubinina, E. A. Kuzmina, L. G. Tomilova, R. B. Vasiliev, A. O. Dudnik, L. I. Ryabova, D. R. Khokhlov

**Affiliations:** 1 Department of Physics, Lomonosov Moscow State University, 1 Leninskie Gory, Moscow, 119991 Russian Federation; 2 Department of Chemistry, Lomonosov Moscow State University, 1 Leninskie Gory, Moscow, 119991 Russian Federation; 3 Institute of Physiologically Active Compounds, Russian Academy of Sciences, 1 Severny proezd, 142432 Chernogolovka, Moscow Region Russian Federation; 4 P.N. Lebedev Physical Institute, Leninskiy prosp. 53, 119991 Moscow, Russia

## Abstract

It is shown that the impedance spectroscopy allows identification of the resistive switching mechanisms in complex composite structures. This statement was demonstrated on an example of organic based sandwich structures with a modified polymer matrix as an active element. The impedance spectroscopy scanning was performed for a series of intermediate states formed within the switching process. Analysis of the experimentally obtained impedance spectra shows that the electron transport is provided by delocalized charge carriers and proceeds via conducting filaments formed in a highly resistive matrix. The filament configuration changes during the switching. With the shift from isolating to conducting states, single isolated filaments are reorganized into a branched network.

## Introduction

Development of fast, compact and low-cost electronic memory elements is one of the priority tasks of the modern microelectronics^1^. Application of the resistive switching effect (RSE) in materials and structures of different types can result in efficient and easy to use memory elements with basic operational parameters that are at least comparable with other competing devices^2,3^. In particular, this refers to devices based on organic materials^1,4–6^. In this case, synthesis can be performed without vacuum technologies, for example from solution by printing^7^. RSE is a drastic resistance change that occurs under application of critical electric field to a sample. RSE based on organic materials is nonvolatile, fast (switching time is less than 10 ns); has 10^5^ cycles endurance, slow degradation and high scalability potential^8^. Moreover a possibility to achieve RSE between more than two states was demonstrated, which allows new possibilities for practical application^9^. In order to optimize the operational parameters, it is essential to understand exact mechanisms of the RSE, which have not been fully understood yet. In this paper, we analyze different RSE stages using the impedance spectroscopy. This method is very efficient for distinguishing contributions in conductivity from various elements in complex structures and monitor their evolution under the influence of external factors^10,11^. Impedance spectroscopy was successfully used for characterization of RSE mechanisms in inorganic materials^12–14^.

## Materials and Methods

We performed RSE studies of materials based on commercially available polymer polystyrene, which is an isolating material and is soluble in organic solvents. As we showed before^8^, composite structures based on polystyrene allow to increase structure conductivity and to essentially reduce RSE voltage without deterioration of other characteristics. It was shown that introduction of nanoparticles into active matrix leads to enhancement of RSE reproducibility and lowers energy consumption^15^. In this paper, a polymer matrix with addition of organic semiconducting particles ((^16Сl^Pc)_3_Lu_2_) and inorganic colloidal CdSe nanoplates (NP) was used as an active element. Mass concentration of (^16Сl^Pc)_3_Lu_2_ and NP was about 55% and 10% respectively. Size of phthalocyanine particles is several angstroms, but particles tend to aggregate into clusters of the size 2–50 nm height and 50–300 nm width^16^. NP have a thickness of 1.2 nm and average lateral dimensions of 100 nm. Folding of nanoparticles into coils of 20–30 nm radius was observed. The size of admixtures does not restrict scaling possibilities of memory elements. The triple-decker lutetium (III) phthalocyanine, bearing electron withdrawing chlorine groups, was chosen due to its high solubility, stability to oxidation and thermal stability^17,18^.

**Figure 1:**
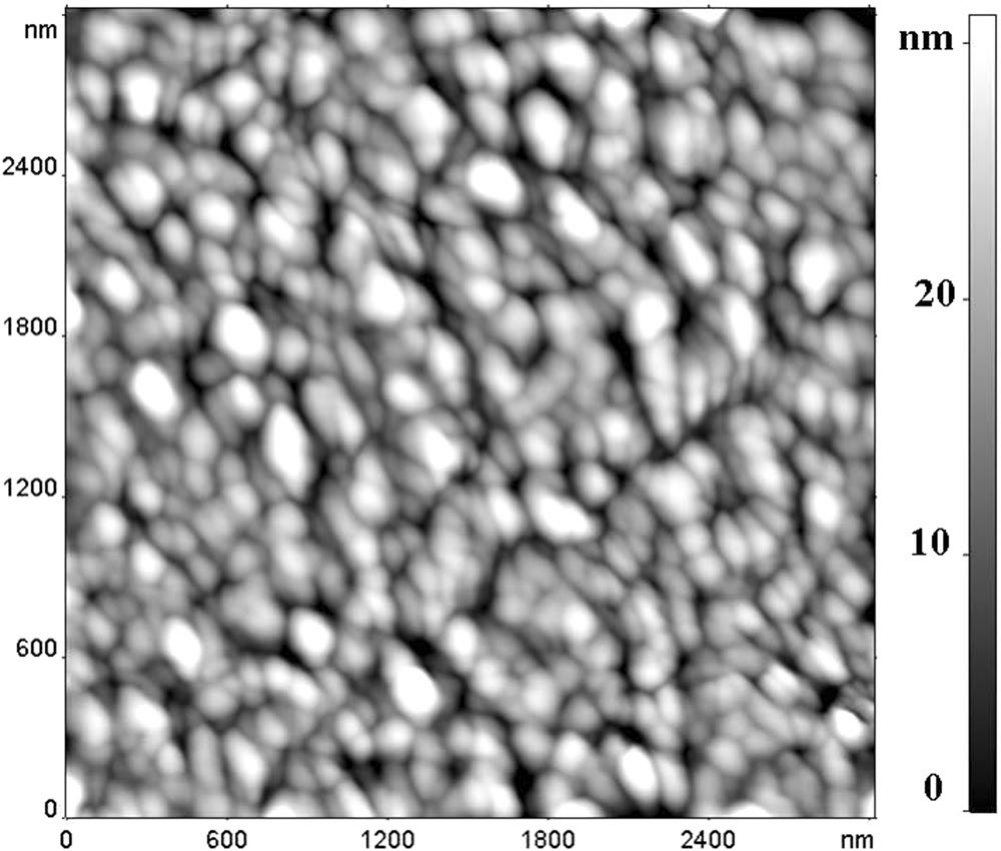
AFM image.

Samples were fabricated in the sandwich geometry. Active layer was deposited on a glass substrate with an ITO conducting layer by drop casting from the solution of a composite material in tetrahydrofuran. Thickness of the active layer was about 2 μm (estimated by optical microscopy). Synthesis of NP is described in the paper^19^, (^16Cl^Pc)_3_Lu_2_ was separated as a by-product in the template synthesis of corresponding monophthalocyanine. Upper contact was formed from the silver paste “Kontaktol”. Sample preparation was carried out at the room temperature in ambient conditions. The homogeneity of active layer was confirmed by morphology studies by AFM (Fig. 1) in the FemtoScan microscope (“Advanced Technologies Center”, Moscow) in the half-contact resonant mode. The resonance frequency used varied between 230–630 kHz, head fpN 20 S was used, tip curvature radius less than 10 nm.

Measurements in DC fields were performed by Keithley 2612A SourceMeter in the range of electrical voltage from 0 to 200 V, scan sweep speed from 0.3 to 3.0 mV/s. Measurements in AC fields were performed by QuadTech 1920 Precision LCR Meter in the frequency range 20 Hz-1 MHz, the signal amplitude did not exceed 2 V and did not exceed switching voltage. The frequency scan step varied according to the frequency range. All measurements were done using the two-probe method. Before the experiment, a standard short and open calibration procedure of the measurement cell unit was performed. All measurements were carried out at 300°K in ambient conditions.

## Results and Discussion

Investigated films are continuous, have granular structure with dense packing of nanometer scale grains (Fig. 1). The maximum height dispersal is less than 50 nm or 2.5% of film thickness.

**Figure 2:**
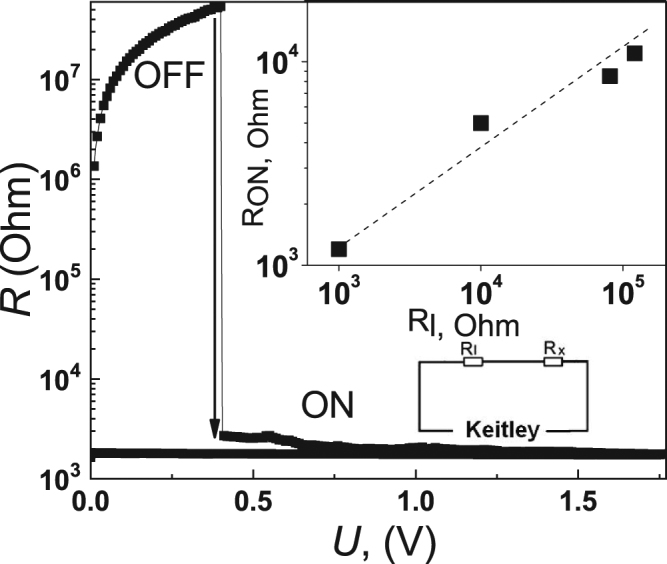
Resistance change at the RSE under external electrical bias. The upper inset shows the R_ON_(R_l_) dependence. Load resistor R_l_ < R_OFF_ allows to perform stabilization of the sample in an intermediate state. The lower inset shows the electrical measurement setup.

The RSE is a reversible transition in structure between states with different conductivity. The first cycle is usually an electroforming procedure with high bias voltage (70 V for the sample described). Further sweeping shows reproducible resistive switching hysteresis. Typical resistances R change at the resistive switching from the low conductive value R_OFF_ to the high conductive R_ON_ is shown in the Fig. 2. Both states are stable after removal of the external electric bias. The critical parameter for the transition into the highly conductive state is the electric field. Introduction of (^16Сl^Pc)_3_Lu_2_ and NP has led to a reduction of critical electric field by a factor of more than 2 compared to the pure polymer sample. The mass concentration of (^16Сl^Pc)_3_Lu_2_ and NP was chosen in order to obtain low switching voltage and high R_OFF_/R_ON_ ratio. Addition of a load resistor R_l_ < R_OFF_ in series with the sample to the measurement circuit allows the voltage to be redistributed between the sample and the load resistor (insert to the Fig. 2, down). During the RSE, the resistance of a sample is reduced, and voltage across it the sample drops down. Consequently, the RSE is not fully completed, and sample resistance is fixed at a value corresponding to an intermediate state between R_OFF_ and R_ON_.

It is possible to obtain a set of intermediate states with different resistance values by varying R_l_ (inset to the Fig. 2, up). The R_ON_(R_l_) states are stable, which allows to carry out measurement cycle in the AC field after removal of the DC voltage.

**Figure 3:**
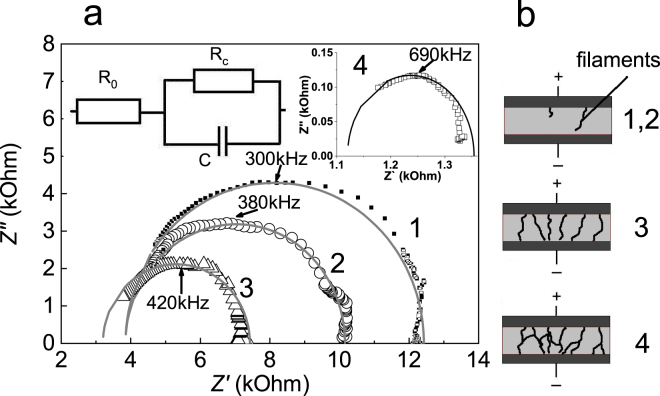
(**a**) Impedance-spectra obtained for intermediate states (numbers near the curves) of the RSE. Symbols- experimental data, curves – fitted dependencies calculated using the equivalent circuit parameters (inset) specified in the Table 1. (**b**) Illustration of the filament formation in intermediate states of the RSE.

For each R_ON_(R_l_) state of the same sample, we performed measurements of the complex impedance Z = Z′ − iZ′′, Z′ is the real part of the total impedance and Z′′ is its imaginary part. Impedance spectra Z′′(Z′) for every R_ON_(R_l_) are single semicircles slightly shifted along the Z′ axis (Fig. 3a, dots). An approximating equivalent scheme of the impedance spectra of such a type is shown in the inset to the Fig. 3a. The equivalent circuit is composed of a resistor R_c_ and a capacitor C connected in parallel, in series with a resistor R_0_. R_0_ can be attributed to the contact resistance. Parameters of the equivalent circuit were calculated from the frequency dependencies Z′′(ω) and Z′(ω) using the following equations:

1 $$\begin{array}{c}{\rm{Z}}^{\prime} ={\rm{R}}/(1+{(\omega {{\rm{R}}}_{{\rm{c}}}{\rm{C}})}^{2})\\ {\rm{Z}}^{\prime\prime} =\omega {{\rm{R}}}_{c}{\rm{C}}{\rm{Z}}^{\prime} .\end{array}$$

Results are shown in the Table 1, corresponding dependencies Z′′(Z′) are depicted as curves in the Fig. 3a. Similar impedance spectra were obtained for the samples with a different particle concentration and different composition. For the sample described in this paper, the RC parameters of intermediate states are in the range of reliable measurements of the impedance setup.

**Table 1:** Fitting parameters, calculated from the frequency dependence of impedance components.

	1	2	3	4
R_l_, kOhm	121	81	10	1
R_с_, kOhm	8.6	6.4	4.2	0.7
R_0_, kOhm	3.8	3.8	3.2	1.6
C, pF	62	66	90	330

Analysis of the impedance spectra allows noting the following. The absence of the Warburg element in the low-frequency region shows that the processes associated with the ionic conductivity do not appear^20^. Particularly, it confirms nearly full desolvation in the structures that were synthesized using solvents. The shape of the impedance hodograph is a semicircle with a good precision. It means that the parameters of the corresponding equivalent circuit (resistor R and capacitor C connected in parallel) do not depend on frequency f. Therefore the real part of the conductance σ’ is also independent on f. According to Mott^21^ in the case of hopping σ’ should change with f variation following the power law σ’ ~ ω^α^, where α is close to unity. Such behavior of σ’ is not present for samples, discussed in this manuscript. Moreover, temperature dependence of the structure resistance is metal like, no activation or hoping is observed. Thus, it can be concluded that the charge transport in the structures studied is determined by delocalized carriers.

As it can be seen from Table 1, R_c_ decreases monotonically with the decrease of the load resistance. The capacitance remains constant for R_l_ ≥ 10 kΩ (curves 1–2 in Fig. 3a). Further conductivity growth results in the capacitance C increase by 50% (curve 3) and by more than 200% (curve 4) of the original value.

Approximation of the dielectric permittivity ε using the plane capacitor equation with the constant C results in the value of ε ~ 3 in intermediate states 1–2, which corresponds to literature values for polystyrene and phthalocyanine complexes^22^. Therefore, no changes of intrinsic composite construction in the 1–2 states that could lead to ε distortion were detected. Additional contributions to capacitance caused by recharging of any centers in this structure may be excluded as well. All states 1–4 exhibit low deviation of conductivity in temperature range 300–77 K and no Arrhenius dependence. In view of all above, it is reasonable to conclude that the charge transport in the structures studied is realized by filamentary conduction of delocalized carriers through isolated metal or carbon filaments^23–25^. In the highest conducting state (4), these channels can widen and overlap. Figure 3b illustrates the process of filament transformation at different stages of the RSE.

It should be noted that in structures with an active element based on organic components, there is no common concept in determining RSE mechanisms. There is a number of alternative models. According to the data presented in review^25^, RSE in polystyrene with organic and inorganic admixtures may be due to formation of conducting filaments, trap recharging and formation of space charge at the interface between a contact and an active layer. Filament formation model is explained by the electric field driven migration of metal nanoclusters from electrodes in dielectric layer. Addition of organic/inorganic particles into polymer dielectric matrix may lead to the local field enhancement and thus lower critical voltages switching^15^.

The structures studied in this paper may be considered essentially as a flat capacitor with a dielectric gap in the R_OFF_ state. In the states with intermediate values of conductivity (Table 1, 1–2 states), capacitive characteristics of the active layer do not change and the conductivity growth can be explained as a leakage, which does not destroy the homogeneity of a dielectric matrix.

Since neither hopping mechanism nor trap recharge were observed, it seems most logical to relate the transport mechanism of delocalized charge carriers to formation of conducting filaments with a total volume not significant compared to the total active layer volume. Transition to the states with lower resistance is accompanied by evolution of isolated conducting filaments into a branchy network. The observed increase in capacitance may be related to the Maxwell-Wagner effect in inhomogeneous media.

A consecutive decrease of the contact resistance R_0_ can be explained by the growth of an intersection area between the filament network and the contact.

Summarizing, we have demonstrated that the impedance spectra measurements of composite organic structures revealing the RSE provide information that determines the application criteria of a particular model in structures of various types. Moreover, the possibility of stabilization of intermediate states in the RSE process is demonstrated, which allows to increase the number of memory states of a memory cell.
